# Neurolymphomatosis detected by ^18^F‐fluorodeoxyglucose‐positron emission tomography

**DOI:** 10.1002/jha2.36

**Published:** 2020-07-07

**Authors:** Yoshikazu Hori, Dai Maruyama, Akiko Miyagi Maeshima, Hanae Ida, Hiroaki Kurihara, Koji Izutsu

**Affiliations:** ^1^ Department of Hematology National Cancer Centre Hospital Tokyo Japan; ^2^ Department of Pathology National Cancer Centre Hospital Tokyo Japan; ^3^ Department of Diagnostic Radiology National Cancer Centre Hospital Tokyo Japan


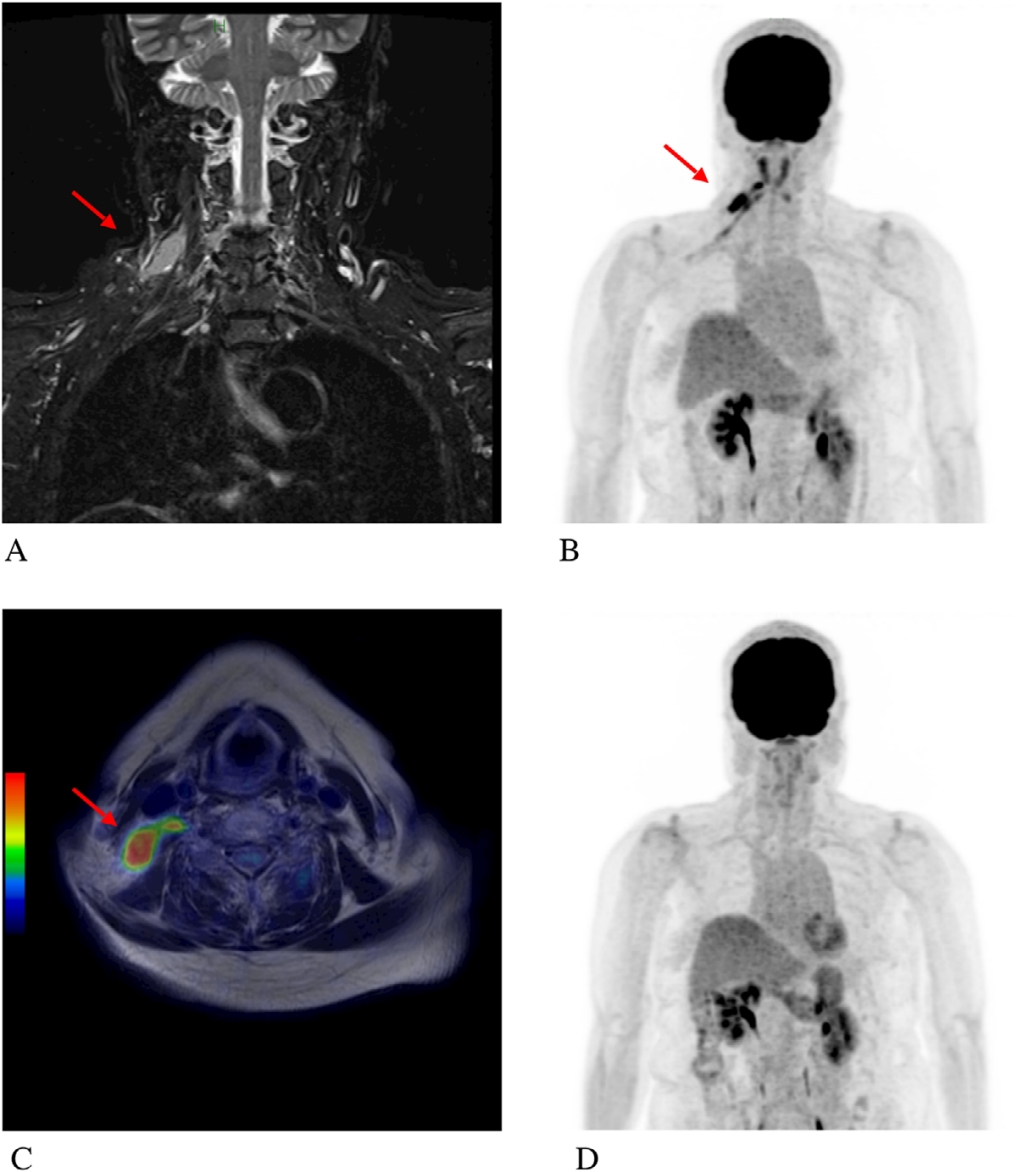


A 75‐year‐old woman was diagnosed with primary breast diffuse large B‐cell lymphoma, non‐germinal centre B‐cell‐like type (stage IE). She was treated using three cycles of rituximab, cyclophosphamide, doxorubicin, vincristine, and prednisolone, followed by radiation therapy (30 Gy/10 fractions). Thereafter, complete metabolic response (CMR) was confirmed by ^18^F‐fluorodeoxyglucose (FDG)‐positron emission tomography (PET)/computed tomography (FDG‐PET/CT). Three months after CMR was confirmed, she developed pain in her right shoulder. Although FDG‐PET/CT was performed, no abnormal findings were noted and she was followed up in an outpatient setting. Six months later, she had difficulty lifting her right upper limb due to pain in her right shoulder. A manual muscle test revealed 2/5 (deltoid), 4/5 (biceps), and weakness of the right upper limb. Coronal short TI inversion recovery magnetic resonance imaging (MRI) demonstrated thickening of the right brachial plexus (Panel A). In addition, abnormal uptake of FDG (SUVmax 12.21) in the right brachial plexus was noted on FDG‐PET/MRI (Panels B and C). No abnormal accumulation of FDG was found in the right breast as a primary involved site and cerebrospinal fluid tests were negative for lymphoma cells. A diagnosis of neurolymphomatosis was made based on her medical history, and MRI and FDG‐PET/MRI findings. Three cycles of high‐dose methotrexate therapy (3.5 g/m^2^) plus rituximab were performed, and CMR was achieved (Panel D).

Neurolymphomatosis is a rare clinical entity defined as infiltration of the peripheral or central nervous system by lymphoma cells, and accounts for 0.2% of all lymphomas. Symptoms vary, and pathological diagnosis is often difficult because biopsy is not always possible such as when the disease site is in an inaccessible area. We should take neurolymphomatosis into consideration when neurological symptoms develop during or after treatment of malignant lymphoma. As demonstrated in the present case, FDG‐PET is a useful diagnostic modality for patients suspected of having neurolymphomatosis; however, caution is needed regarding the possibility of false‐negative FDG‐PET results, especially in the early stage of neurolymphomatosis.

